# Localizing triplet periodicity in DNA and cDNA sequences

**DOI:** 10.1186/1471-2105-11-550

**Published:** 2010-11-08

**Authors:** Liya Wang, Lincoln D Stein

**Affiliations:** 1 Cold Spring Harbor Laboratory, Williams #5, Cold Spring Harbor, NY, 11724, USA; 2 Ontario Institute for Cancer Research, 101 College St., Suite 800, Toronto, ON, M5G0A3, Canada

## Abstract

**Background:**

The protein-coding regions (coding exons) of a DNA sequence exhibit a triplet periodicity (TP) due to fact that coding exons contain a series of three nucleotide codons that encode specific amino acid residues. Such periodicity is usually not observed in introns and intergenic regions. If a DNA sequence is divided into small segments and a Fourier Transform is applied on each segment, a strong peak at frequency 1/3 is typically observed in the Fourier spectrum of coding segments, but not in non-coding regions. This property has been used in identifying the locations of protein-coding genes in unannotated sequence. The method is fast and requires no training. However, the need to compute the Fourier Transform across a segment (window) of arbitrary size affects the accuracy with which one can localize TP boundaries. Here, we report a technique that provides higher-resolution identification of these boundaries, and use the technique to explore the biological correlates of TP regions in the genome of the model organism *C. elegans*.

**Results:**

Using both simulated TP signals and the real *C. elegans *sequence F56F11 as an example, we demonstrate that, (1) Modified Wavelet Transform (MWT) can better define the boundary of TP region than the conventional Short Time Fourier Transform (STFT); (2) The scale parameter (a) of MWT determines the precision of TP boundary localization: bigger values of a give sharper TP boundaries but result in a lower signal to noise ratio; (3) RNA splicing sites have weaker TP signals than coding region; (4) TP signals in coding region can be destroyed or recovered by frame-shift mutations; (5) 6 bp periodicities in introns and intergenic region can generate false positive signals and it can be removed with 6 bp MWT.

**Conclusions:**

MWT can provide more precise TP boundaries than STFT and the boundaries can be further refined by bigger scale MWT. Subtraction of 6 bp periodicity signals reduces the number of false positives. Experimentally-introduced frame-shift mutations help recover TP signal that have been lost by possible ancient frame-shifts. More importantly, TP signal has the potential to be used to detect the splice junctions in fully spliced mRNA sequence.

## Background

Most protein-coding genes in eukaryotic cells are composed of alternating introns and exons. With the exception of the extreme 5' and 3' ends of the first and last exons, which contain un-translated regions (UTRs), exons encode protein sequence. These are so-called "coding designated segments" or CDS. Combination of any three nucleotide bases is a codon that encodes one amino acid residue (except three stop codons). CDS regions are known to exhibit a periodic organization of three bases, a triplet periodicity (TP) [1], which is generally considered a good preliminary indicator of protein-coding exon locations. TP based approaches for prediction of CDS [2-6] is model-independent since it does not need to be trained. On the other hand, model-based methods, like Markov chains [7], tend to be more precise by training a supervised classifier based on database of previously known organisms' genomic information, though it is usually not trivial to construct negative samples for training purposes. However, when sequenced organisms have coding segments that are not represented in the currently available database, TP based methods may complement model-based systems.

TP signals can be detected by applying windowed Fourier Transform, also called Short Time Fourier Transform (STFT), along DNA sequences [2]. However, the ability of the STFT to identify the precise boundaries of the TP signal is limited by its requirement of an arbitrarily chosen window size over which the spectrum is calculated. It has been shown that the choice of different window lengths directly affects the prediction accuracy [8]. The window size problem of STFT is the result of the resolution tradeoff between the time and frequency domains: FT is applied to the data from time zero to end with high resolution in the frequency domain but none in time domain; STFT (or windowed FT) captures the time domain resolution but loses some resolution in the frequency domain. A natural improvement on STFT is the wavelet transform (WT) that allows one to balance resolution at any time and frequency [9]. However direct application of WT is limited by the fact that the coding regions with TP will present the same frequency (1/3) under different scales [5]. For this reason, the ability of WT to automatically capture different frequencies is not appropriate here. Here we used a Modified Wavelet Transform (MWT) algorithm to show: (1) how TP boundary can be defined with greater accuracy; (2) how the TP profile can be used to infer the splice junctions in mature mRNA sequences; (3) how ancient frame-shift mutations may explain the loss of TP signals in coding regions; and (4) how the 6 bp periodicity in some intronic and intragenic regions can be identified and corrected in order to reduce false positive identifications of coding regions.

## Methods

### DNA sequence data and simulated sequence data

For a biological test case, we used the sequenced contig F56F11 of *C. elegans*, a 43 kbp sequence containing six protein-coding genes and two non-coding genes. The 8000 bp-long gene F56F11.4 which starts at contig position 7021 (GenBank access number AF0099922, positions 7021-15020) has been used extensively as a test case for TP detection techniques [5,6,10]. This gene contains five CDS of length greater than 100 bp located at positions 928-1039, 2528-2857, 4114-4377, 5465-5644, and 7255-7605 relative to the start of the gene (a short alternatively-spliced sixth exon at the extreme 5' end contains a coding region that is too short to be analyzed by these techniques).

In addition to the real test case, we used following simulated sequence of length 900 with exact periodicities (p = 3, 6 or 9).

(1) $$\text{u}\left[\text{n}\right]=\{\begin{array}{ccc} 0 & , & \begin{array}{cccc} \text{if} & \text{n}<300 & \text{or} & \text{n}>600 \end{array}\\ 1 & , & \begin{array}{ccc} \text{elseif} & \text{n}=300+\text{m}*\text{p} & \end{array}\\ 0 & , & \text{otherwise} \end{array}$$

Where m is a non-negative integer and different p values simulates period of 3, 6 or 9 patterns from base position 300 to 600.

### Short time Fourier transform

In the results section, we compare the performance between STFT and MWT. Before performing STFT or MWT, the DNA sequence is numerically mapped to four binary indicator sequences for bases A, C, G, and T. If a base type (e.g. A) presents at base position k of the DNA sequence, we put a '1' at position k of this base's binary indicator sequence, otherwise, we put a '0' there. E.g. AGTCA becomes the four binary strings 10001 for the A bases, 00010 for the C bases, 01000 for G and 00100 for T. The four binary indicator sequences have the same length as the original DNA sequence. We use u_A_, u_C_, u_G_, and u_T _to represent these four binary sequences, and apply Fourier Transform to them separately to get a new sequence of U[k] with the same length as the original.

(2) $$\text{U}\left[\text{k}\right]=\sum_{\text{n}=\text{0}}^{\text{N}-\text{1}}{\text{u[n]e}}^{-\text{j}\frac{\text{2}\pi }{\text{N}}\text{kn}}\begin{array}{ll} , & \text{k}=\text{0,1,}...\text{N}-\text{1} \end{array}$$

The sequence U[k] provides a measure of the frequency content at 'frequency' k, which corresponds to an underlying period of N/k samples. The power spectral density (PSD) is defined as,

(3) $$\begin{array}{l} \text{PSD}\left[\text{k}\right]=\\ \begin{array}{c} |{\text{U}}_{\text{A}}\left[\text{k}\right]{|}^{2}+|{\text{U}}_{\text{C}}\left[\text{k}\right]{|}^{2}+|{\text{U}}_{\text{G}}\left[\text{k}\right]{|}^{2}+|{\text{U}}_{\text{T}}\left[\text{k}\right]{|}^{2} \end{array} \end{array}$$

We choose a window size N which is a multiple of p, and slide it across the DNA sequence. The PSD[N/p] value given by STFT is recorded for each base located at the center of the sliding window. The TP property of a DNA sequence implies that PSD[N/3] (when p = 3) values peak in coding regions. Thus the plot of PSD[N/3] against base position will distinguish CDS from non-coding regions. Later we will refer PSD[N/3] simply as PSD for each base position.

### Modified wavelet transform

A continuous wavelet transform of a continuous, square-integrable function u(*x*) at a scale a > 0 and position k is expressed by the following integral.

(4) $$\text{U}\left(\text{a},\text{k}\right)=\frac{\text{1}}{\sqrt{\text{a}}}\int \text{u}\left(\text{x}\right)\psi^{*}\left(\frac{\text{x}-\text{k}}{\text{a}}\right)\text{dx}$$

Where * means complex conjugate. Let t = (x - k)/a, then ψ(t) is a continuous function in both the time domain and the frequency domain called the wavelet function. One of the wavelet functions, the complex Morlet wavelet function is defined by following equation [11].

(5) $$\begin{array}{c} \psi \left(\text{t}\right)={\text{e}}^{-{\text{t}}^{\text{2}}/2}\left(\text{cos}\omega_{\text{0}}\text{t}+\text{jsin}\omega_{\text{0}}\text{t}\right)\\ ={\text{e}}^{-{\text{t}}^{2}/2}{\text{e}}^{\text{j}\omega_{0}\text{t}} \end{array}$$

Where ω_0 _is called the basic frequency of Morlet wavelet function. For constructing MWT, scale parameter a is added to Morlet wavelet function and now it becomes.

(6) $$\psi \left(\text{t}\right)={\text{e}}^{-{\text{t}}^{2}/2}{\text{e}}^{\text{ja}\omega_{0}\text{t}}$$

Such modification ensures that the frequency part (${\text{e}}^{\text{ja}\omega_{0}\text{t}}$) is independent of scale parameter a since t = (x - k)/a. For practical reason, the length (or vanishing moments) of the wavelet analysis function (N) is taken as 1200 unless specified: a longer length will give higher precision but takes longer to compute. Now if ω_0 _is taken as N/b, it can precisely capture b base periodicity. The normalization factor $\text{1}/\sqrt{\text{a}}$ in equation (4) is critical to ensure a constant norm in the space *L*^2^(*R*) of square integrable functions, which in turn ensures equal areas under the MWT for two equal-amplitude Fourier components in the power spectrum. Without the normalization factor, MWT is similar to the modified Gabor Transform [5]. PSD(a, b) can be calculated by equation (3) after obtaining U by equation (4) to capture b base periodicity under scale a. b is chosen as 3 for capturing TP signal and a is 5 unless specified. Later we will also refer PSD(a, b) simply as PSD for each base position.

## Results

### The effect of the scale parameter

**Figure 1:**
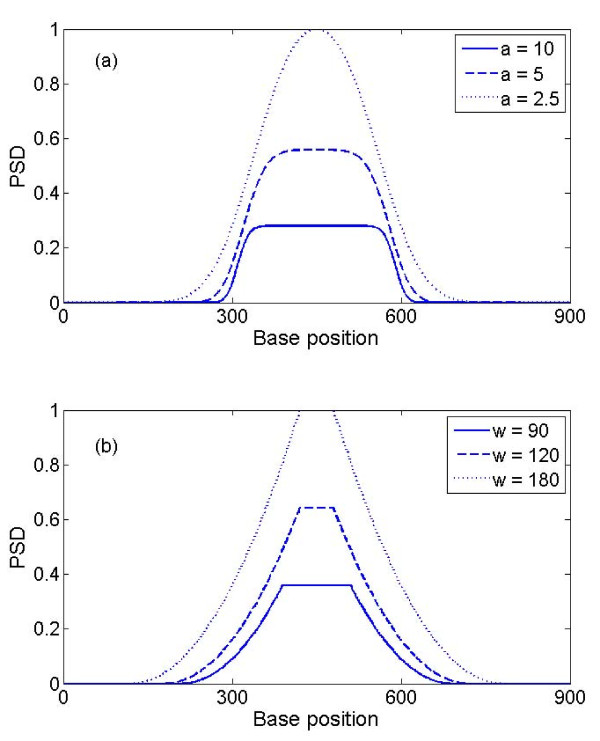
**PSD plot of simulated data with p = 3**. (a) Comparison of different scales for MWT; (b) Comparison of different window sizes for STFT. PSD values are normalized by dividing the maximal value under scale 2.5 for MWT in (a), or the maximal value under window size 180 in (b)

Using the simulated data with triplet periodicity (p = 3 in equation (1)), we compared the performance of MWT (Figure 1a) and STFT (Figure 1b). Given that TP in simulated signal starts at 300 and ends at 600, in general, the MWT produces a sharper boundary between coding and non-coding segments than STFT, thus better accuracy if we choose a cut-off PSD threshold of 0.2 (regions with PSD >0.2 are scored as coding regions). The amplitude of the PSD curve is strongly dependent on the choice of window size in the STFT algorithm, and of the scale parameter in the MWT algorithm. If we again choose a cut-off threshold of 0.2, it can be seen from Figure 1 that STFT defined boundary is markedly sensitive to changes in window size, whereas MWT defined boundary is more robust to the choice of scale parameter. Furthermore, due to the sharpness of the MWT-generated PSD curve, the choice of cut-off has less of an effect on the inferred location of the protein-coding boundary in MWT than in STFT.

From Figure 1(a), it can be seen that MWT with larger scale parameters gives a sharper boundary but with a lower peak amplitude, making it less tolerant to noise. For real DNA sequences, larger scale parameters (equivalent to a smaller window size) will result in more noise in introns and intergenetic regions since smoothing is performed over smaller region. A hybrid approach can be taken by using a smaller scale parameter to locate candidate coding regions and then refining the boundaries of these regions by re-running the MWT with a larger value of scale. Combining this technique with the well known GT-AG rule, which governs the great majority of Type I eukaryotic splice junctions [12], and open reading frame identification, might enable precise identification of the CDS boundaries.

### TP signal in a real biological sequence

**Figure 2:**
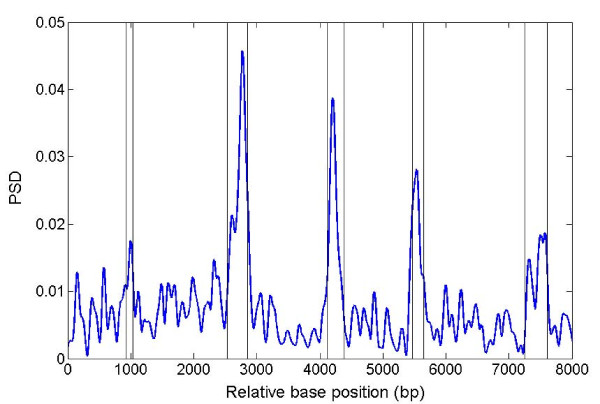
**PSD plot of sequence F56F11.4**. The vertical lines are drawn on the splice junctions.

Figure 2 shows the PSD plot across gene F56F11.4 using MWT. It can be seen that five exon regions are correctly identified except that the first (most left) peak is relatively weak due to the relatively short exon length (112 bp).

**Figure 3:**
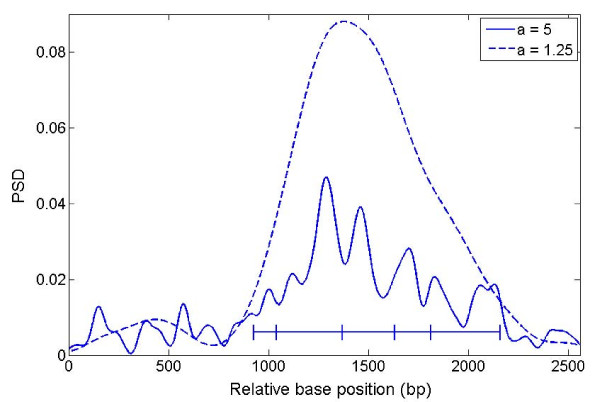
**PSD plot of sequence F56F11.4 without introns**. PSD plot at two different scales, 5 and 1.25 for MWT. The line segments on the bottom show the splice junctions.

After removing the introns of gene F56F11.4, we merged the exons and plotted PSD under two different choices of scale parameter (Figure 3). The plots show a dramatic increase in PSD at the transition between non-coding and coding sequence. At a scale of 1.25, the PSD plot can clearly distinguish the 5' and 3' UTRs from the coding region. However, under scale of 5, the PSD plot gives a better indication of TP boundary on both sides for dividing 5' UTR and first exon, or last exon and 3' UTR. In practive, larger values of the scale parameter have a higher resolution for revealing details hidden within the broad PSD peak obtained under smaller scales. In Figure 3, the horizontal line represents the coding region with vertical lines marking the boundaries of individual exons.

More interestingly, Figure 3 shows that, under the larger value of scale (a = 5), individual exons can be distinguished from each other even when they are merged together as they would appear in fully-spliced mRNA. The exceptions are the second and last exons, each of which contains two distinct PSD peaks (Figure 2 and Figure 3).

**Figure 4:**
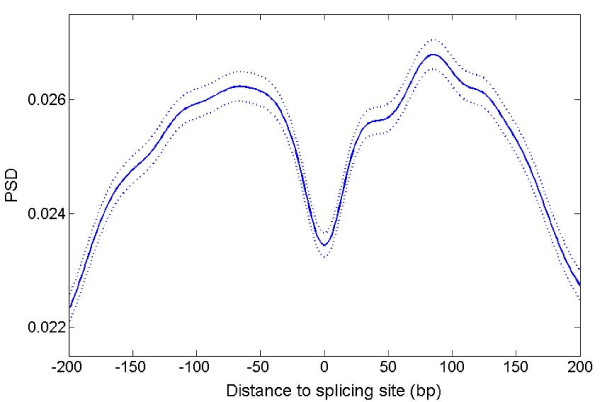
**PSD plot of two adjacent exons**. Solid line shows the average of PSD values of 4655 adjacent exon pairs each longer than 150 bp. Dashed line shows plot with +/- standard errors. The length of the wavelet analysis function (N) is taken as 512 and scale parameter a is taken as 5.

To confirm that the diminution of the TP signal at splice junctions is a general phenomenon, we extracted 4655 adjacent *C. elegans *exon pairs from WormBase version WS211. Because of the length limitation on TP determination, we restricted the pairs to those in which the exons on both sides of the splice site were greater than 150 bp; we also eliminated pairs involving the first or last exon of each gene in order to avoid possible effects of UTRs. The average and standard error of the 4655 PSD plots across +/- 200 bp around the splicing site (position zero) are shown in Figure 4. This confirms that the drop of TP signal around the splicing site is a general phenomenon. This finding suggests that the TP signal could potentially be used to infer the splicing site from fully spliced mRNA sequences, and might be a useful criterion to enhance the accuracy of software that aligns cDNA sequence to the genome.

The most straightforward explanation for this result is that the diminution of TP signal around splice junctions is due to the different TP patterns between adjacent exons. The presence of splicing cis-regulatory elements in the exon, such as exonic splice enhancers [13], which locally distort the pattern of evolutionary constraint, might contribute to the dimunution. However, the valleys are still observed after removing up to 90 bp on each side of the splice junction (Additional file 1, Figure S1), suggesting that these elements, if present, are not the dominating factors.

### The effect of 'frame-shift' mutations

A frame-shift mutation is a genetic mutation caused by insertion or deletion of 3n + 1 or 3n + 2 nucleotides from the coding region of a DNA sequence. Such mutations change the downstream codons which in turn changes the final protein product [14,15]. Such mutations will break the TP pattern within the exon by altering the phase of the codon bias around the mutation point. Here we examined whether the TP signal can be used to detect frame-shift mutations and vice versa.

**Figure 5:**
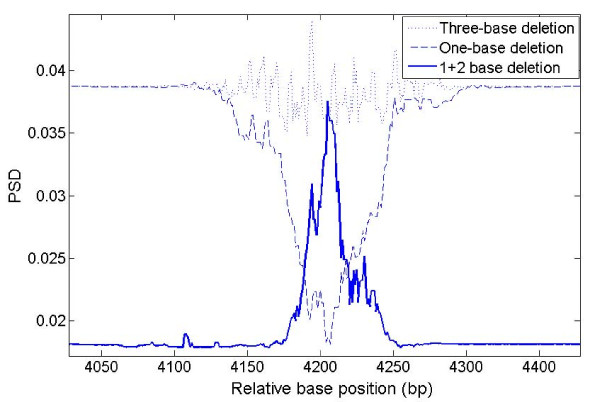
**Amplitude of PSD peak under various base deletions**. Third PSD peak in Figure 2 is investigated for comparison of amplitude under 3-base deletion, 1-base deletion, and 1-base deletion followed by 2-base deletion at various base positions.

Using the PSD peak corresponding to the third exon of gene F56F11.4, we showed the effects of base deletions at various positions in Figure 5, and the effects of different base insertion after a C nucleotide deletion in Additional file 1, Figure S2. Figure 5 shows that losing three consecutive nucleotides only slightly changes the amplitude of the peak. However, a one-base deletion at a certain range can dramatically reduce the amplitude of the peak. A subsequent compensatory two-base deletion, on the contrary, will restore the peak (also shown in Additional file 1, Figure S3). On the other hand, the TP signal is not sensitive to single nucleotide variations (Additional file 1, Figure S2). Here the PSD plots are generated by re-calculating TP after a mutation at a site and recording the maximal peak height. In Additional file 1, Table S1, we randomly pick 45 human exons and provide the summary for likely evolutionary frame-shifts.

**Figure 6:**
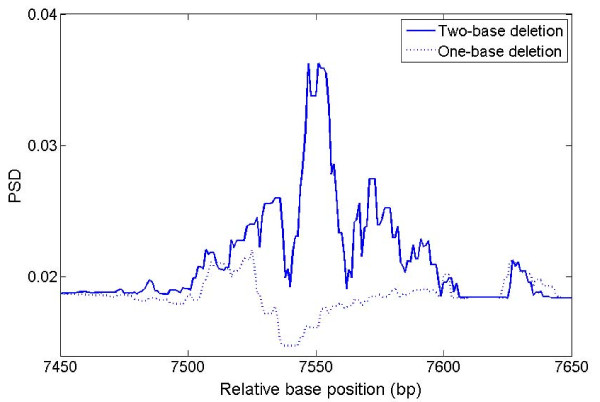
**Amplitude of PSD peak under various base deletions**. Fifth PSD peak in Figure 2 is investigated for comparison of the amplitude under 1-base deletion or 2-base deletion at various positions.

During our examination of F56F11.4, we noted that the last exon of gene is the longest one, but does not have the expected strongest PSD peak (since PSD signal is cumulative). We suspected that this might reflect an ancient frameshift mutation (or more than one insertion/deletion). To investigate this possibility, we introduced a series of one- and two-base deletions at various positions around the last exon of F56F11.4. The PSD plot in Figure 6 shows that a two-base deletion around position 7550 greatly enhances the TP signal (also shown in Additional file 1, Figure S4). Comparing Additional file 1, Figure S4 with Figure 2, it can be seen that the amplitude of the last PSD peak is greatly increased. In Additional file 1, Figure S5, we show that the TP signal can be enhanced for an exon of adjacent gene F56F11.3 by using combinations of one- and two-base deletions at two different base positions.

Interestingly, nucleotide alignments between the *C. elegans *genome and related nematode species [16] detects indels around these regions (Additional file 1, Figure S4 and Additional file 1, Figure S5), providing independent support for ancient frameshift mutations at these sites.

### The effect of 6 bp periodicity

It has been pointed out that p bp periodicity peak will be observed in the power spectrum even if the sequence only has 2p bp periodicity when Fourier Transform is used [17]. This is an inherent character of FT and MWT since, for example, if there is a peak at k = N/6, there will be a peak at k = N/3 using equation (2) or (4). This drawback needs to be corrected when either FT or MWT is applied to the identification of DNA triplet periodicity.

**Figure 7:**
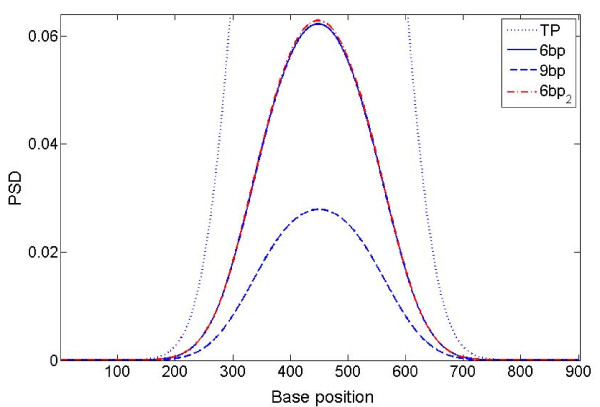
**PSD plot of different periodicities**. PSD plot of simulated signal with p = 3 (dotted line), p = 6 (solid line) and p = 9 (dashed line) generated by MWT(b = 3), and PSD plot of simulated signal with p = 6 (red dotted dashed line) generated by MWT(b = 6).

To show that 6 bp or 9 bp periodicity can be captured by MWT with b = 3 for TP, we generated three simulated sequences with p = 3, 6, or 9 using equation (1). Figure 7 shows the PSD signal captured by MWT with b = 3. The amplitude of the peak drops for larger p since we fixed the length of the sequence tested for periodicity. In theory, the amplitude of the peak for the same length sequence with only 6 bp or 9 bp periodicity will be 1/4 and 1/9 of that for 3 bp periodicity. For example, the number of repeats of 6 bp is 1/2 that of TP (e.g. sequence 100100100100 has 4 repeats of 100 while sequence 100000100000 has only 2 repeats of 100000) given the same sequence length, and the ratio becomes 1/4 when the power of 2 is taken when computing the power spectrum (equation (3)). The simulated signal indeed shows an approximate peak height of 0.25 (TP), 0.062 (6 bp), and 0.027 (9 bp). By setting b = 6 instead of 3, MWT will capture the 6 bp periodicity instead of 3 bp periodicity. The PSD plot for a periodicity of 6 of the simulated data is shown in Figure 7 (red dashed dot line and labelled as 6 bp_2_); note that the peaks have almost same amplitude as that generated by MWT with b = 3.

**Figure 8:**
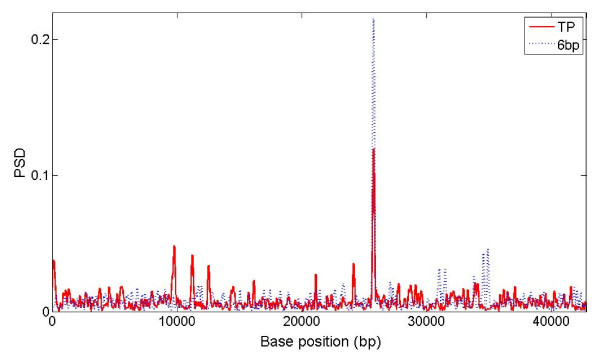
**Effect of 6 bp periodicity**. PSD plot of sequence F56F11 of *C. elegans *generated by MWT(b = 3, TP) and by MWT(b = 6, 6 bp). Scale of 2.5 is used.

Figure 8 demonstrates an extreme case within the *C. elegans *sequence F56F11. There is a strong PSD peak (red solid line) around base 25650-25850 for TP but it is not defined as a CDS in the NCBI annotations. The PSD plot generated by MWT with b = 6 (dashed line) shows a very strong peak at the same location; after subtracting the b = 6 signal from the b = 3 signal, this false positive TP peak gets eliminated.

## Discussion

Several hypotheses have been advanced to explain the origin of the TP in coding sequence [10,18,19]. The simplest explanation is codon bias, which increases the probability that nucleotide triplets will appear in the same phase, but the matter is far from settled. Further it remains unclear why some CDS do not have apparent TP signal, and why some non-CDS segments have strong TP signals. Our analysis provides useful insights into TP properties at both the algorithmic and biological perspecives. It shows that MWT can better precisely delimit coding boundaries than STFT, and that a simple procedure of introducing artificial frame-shift mutations into protein-coding candidate regions can recover signal that was lost by presumptive ancient frame-shift mutations. While, this step might end up identifying regions that have lost coding capability during evolution, the identification of ancient coding sequences may still be of interest for comparative genomics.

In and of itself, the TP property is inadequate for gene prediction. However, it may be a useful adjunct to other techniques, particularly in the identification of protein-coding genes that are unusual in one way or another. For example, a recent study [20] showed that highly tissue-specific protein coding exons can be discovered via massive RNA-sequencing of 69 lymphoblastoid cell lines derived from unrelated Nigerian individuals that were not predicted by conventional model-based gene prediction algorithms. One can speculate that some of these exons remained undiscovered because they depart from the expectations of the model. Protein coding segment prediction methods based on the model-independent TP property would provide a valuable complement to the conventional gene prediction algorithms.

Using simulated and real life sequencing data, we demonstrated that the MWT scale parameter can be reduced to give more robust prediction of broad TP regions or increased to provide higher resolution of the TP boundary. One way to achieve both will be running MWT with a smaller scale to identify TP regions followed by re-running the algorithm using larger values of the scale. Though it is not clear whether the edges of TP are completely consistent with the edges of protein coding regions (exons), TP edges could be further refined by known knowledge, such as GT-AG rule if done carefully.

**Figure 9:**
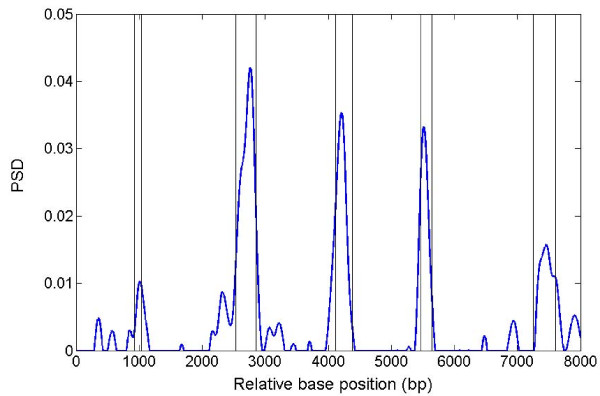
**PSD plot of sequence F56F11.4 after subtracting 6 bp effect. **After subtraction, negative values are changed to zero. Scale of 2.5 is used for MWT.

A drawback of MWT is that the signal obtained for a periodicity of 3 overlaps with the 6 and 9 bp periodicity, which might be contained in introns and intergenic regions, especially when these regions are very long. Using simulated data, we showed that the 6 bp effect can be estimated by MWT with b = 6 and then subtracted from the b = 3 profile. After setting negative peaks to zero, the PSD plot of F56F11.4 (Figure 9) shows that noise is suppressed and real exon peaks are retained. Though not shown, the subtraction also removed the big false positive peak around base 25650-25850 for sequence F56F11. Alternatively, the 6 bp effect can also be used as a control for confirmation of true positives instead of direct subtraction. It is worthwhile to mention that a 9 bp periodicity can cause false positives as well but the chance is much lower given that same length sequence with 9 bp periodicity only contributes 1/9 the amplitude levels compared with those with TP. The subtraction of 6 bp and 9 bp periodicity effects could be substantial for species with much longer introns, as shown in Additional file 1, Figure S6.

The most interesting result stemming from this analysis is the diminishing TP signal around the splice junction in mature mRNA sequences. The valley remains after deleting up to 90 bp of sequence immediately up and downstream of the junction. This implies that an extended region surrounding the splice site is under a different set of evolutionary constraints which reduces the TP signal. An alternative hypothesis is that areas of weak TP signal are favoured sites for intron birth. It is interesting to note that Additional file 1, Figure S4 shows that the last exon of gene F56F11.4 contains two different TP patterns even after making artificial frame-shift mutations. Perhaps this region once had an intron and lost it; alternatively this region might have an increased probability of acquiring an intron over the course of future evolution. In support of the first hypothesis, we note that there is a 657 bp deletion at the site of the TP valley in *C. elegans *relative to *P. pacificus *(Additional file 1, Figure S4), suggesting the presence of an intron in the common ancestor of these two nematodes.

## Conclusions

MWT is a promising method for capturing triplet periodicity in DNA sequence. Artificial 'frame-shift' mutations and correction for the effect of the 6 bp periodicity signal could further improve the prediction. We also hypothesize that TP property of exons might carry evolution evidence about frame-shift mutations and the separation of exons by introns.

## Authors' contributions

LW carried out the work and drafted the manuscript with LDS. Both authors have read and approved the final manuscript.

## Supplementary Material

Additional file 1 **Supporting materials**. This file contains Figure S1 to S6 and Table S1. Click here for file
