# Supplementary material for: Localizing triplet periodicity in DNA and cDNA sequences
Source: BMC Bioinformatics. 2010 Nov 8;11:550. doi: 10.1186/1471-2105-11-550 (PMC2992068; doi:10.1186/1471-2105-11-550)
Supplement: Additional file 1 — Supporting materials. This file contains Figure S1 to S6 and Table S1. [file 1471-2105-11-550-S1.PDF]

Supporting materials

Figure S1. PSD plots of two adjacent exons.

Each line shows the average of PSD values of 4655 adjacent exon pairs each longer than 150 bp with various number of base pairs removed on both sides of the splicing sites. The length of the wavelet analysis function (*N*) is taken as 512 and scale parameter a is taken as 5. Various number of bases are deleted on both side the splice junctions.

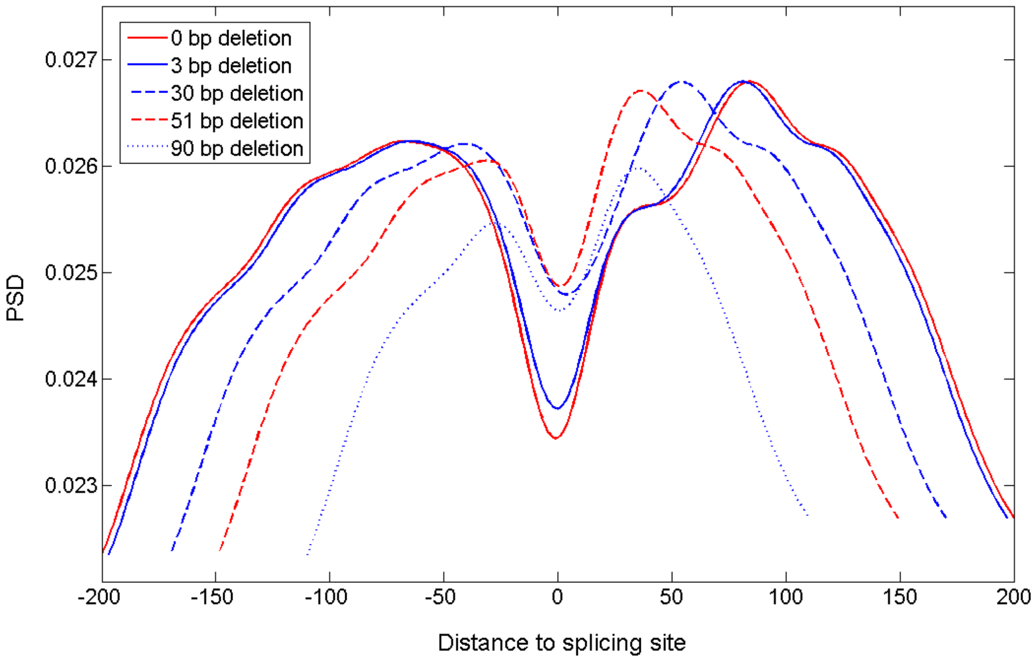

Figure S2 - Amplitude of PSD peak under various insertions

Third PSD peak in Figure 2 is investigated for comparison of amplitude under 1-base insertion of A, G, T at various positions after one base (C) deletion at position 4208.

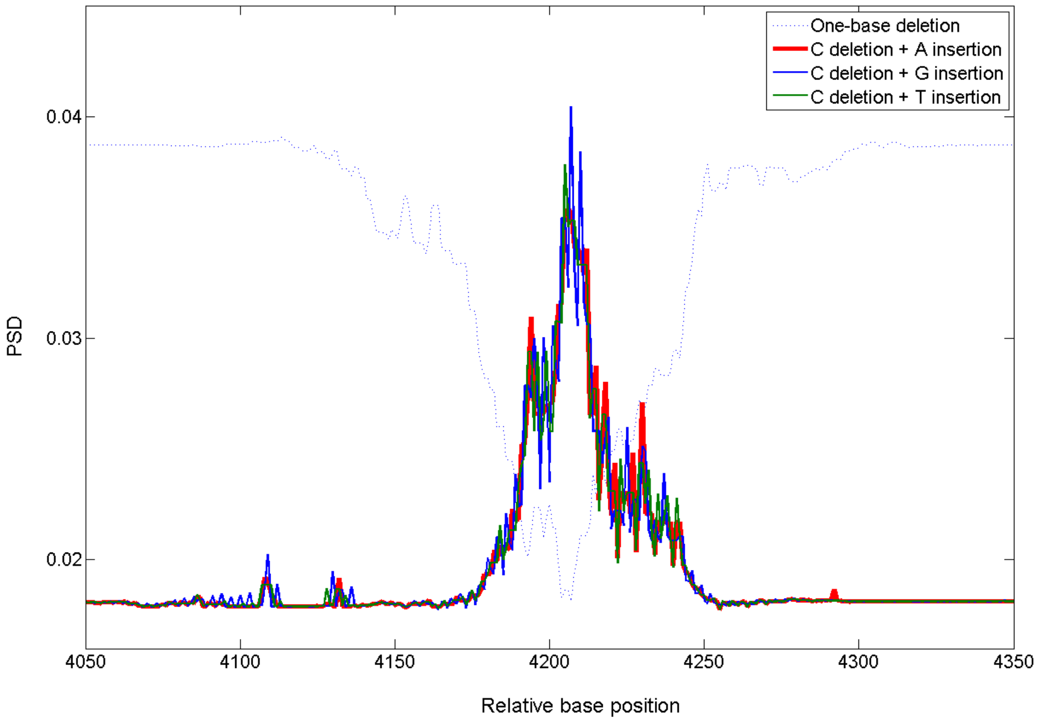

Figure S3. Amplitude of PSD peak under various base deletions

The second exon in Figure S6.3 is investigated for comparison of amplitude under 3-base deletion at various base positions with 1-base deletion, and 1-base deletion followed by 2-base deletion. The base position is relative to 7001 for sequence X78212.1 (H.sapiens diamine oxidase gene).

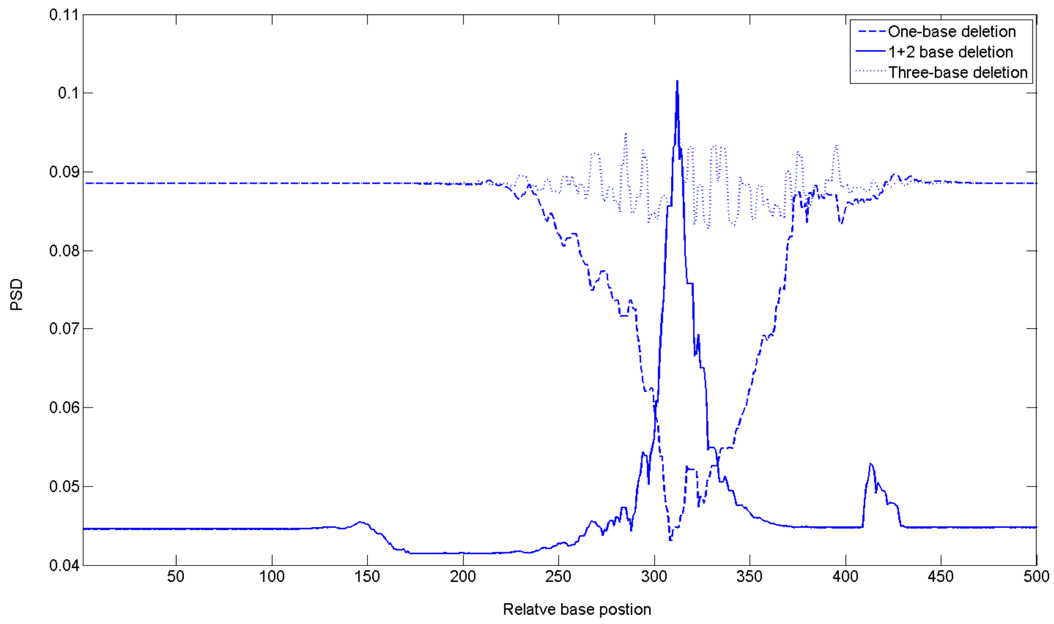

Figure S4 - Reconstructed power spectrum plot

Reconstruction of the PSD plot in Figure 2 after 2-base deletion at position 7547-7548.

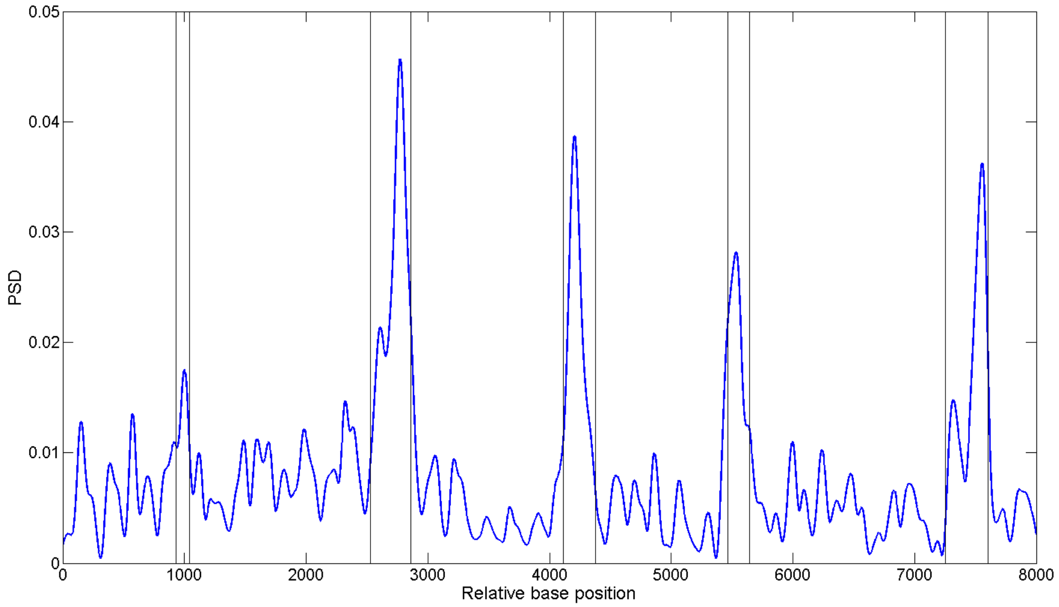

The sequence before and after deletion (in red) are shown below with coded amino acid.

↓

GTTGGAAAAGTGATGCAGAAGGATTCGGAAAAGAATATGTCGATTAAGAAGTTGTGGAAATAA

V G K V M Q K D S E K N M S I K K L W K

GTTGGAAGTGATGCAGAAGGATTCGGAAAAGAATATGTCGATTAAGAAGTTGTGGAAATA

V G S D A E G F G K E Y L D *

Interestingly there are 657 bp deletion right after the deleted AA (pointed by arrow) when *C. elegans* is aligned with *p_pacificus*, as seen in UCSC genome browser. But Figure 6 also show that two base deletion at various positions can also double the peak height. It is the downstream sequence that really got changed.

However, the 657 bp deletion might also be the reason that the last exon in Figure S4 has two peaks, which is that an intron has been lost during evolution. Since Figure 4 and Figure S1 both demonstrate that adjacent exons have different TP patterns which results in two distinguishable peaks.

Figure S5. PSD plot for sequence F56F11.3 of *C. elegans* (27001-28400). Subsequent positions are relative to 27001. (a) Amplitude of PSD peaks (dotted line) in panel (b) under 1-base deletion or 2-base deletion at various positions; (b) Comparison of original peak with reconstructed peak after 2-base deletion at position 764-765 followed by 1-base deletion at position 717.

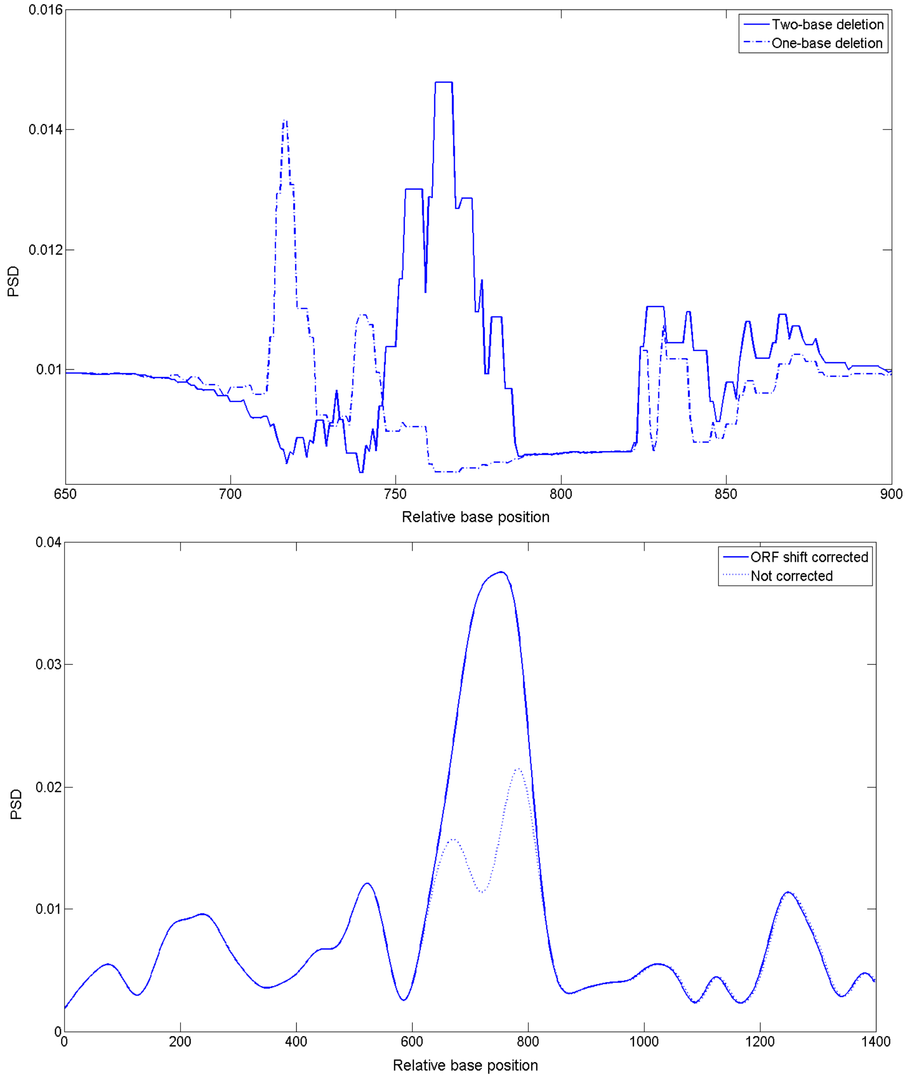

Figure S5 showed the new PSD plot if we make two base deletions at position 27764 and 27765, followed by one base deletion at position 27717. Following is the sequence before and after deletion (in red) with coded amino acid. There is also 12 bp deletion between the downstream AG and GA (pointed by arrow) when *C. elegans* is aligned with *c_remane*, *C_briggsae*, and all others except *p_pacificus* (no data).

CTGATTCATTCGACTTCAAATTTCGATATCGCTTCCTCGAGCTCCGGAGATTCAGGCCATCAGGAT

L I H S T S N F D I A S S S S G D S G H Q D

CTGATTATTCGACTTCAAATTTCGATATCGCTTCCTCGAGCTCCGGAGATTCGCCATCAGGATCAT

L I I R L Q I S I S L P R A P E I R H Q D H

Figure S6. PSD plot for various genes. (a) PSD plot with horizontal bar labeling two coding exons; (b) PSD plot after subtracting the effect of 6bp periodicity; (c) PSD plot after subtracting the effects of both 6bp and 9bp periodicities.

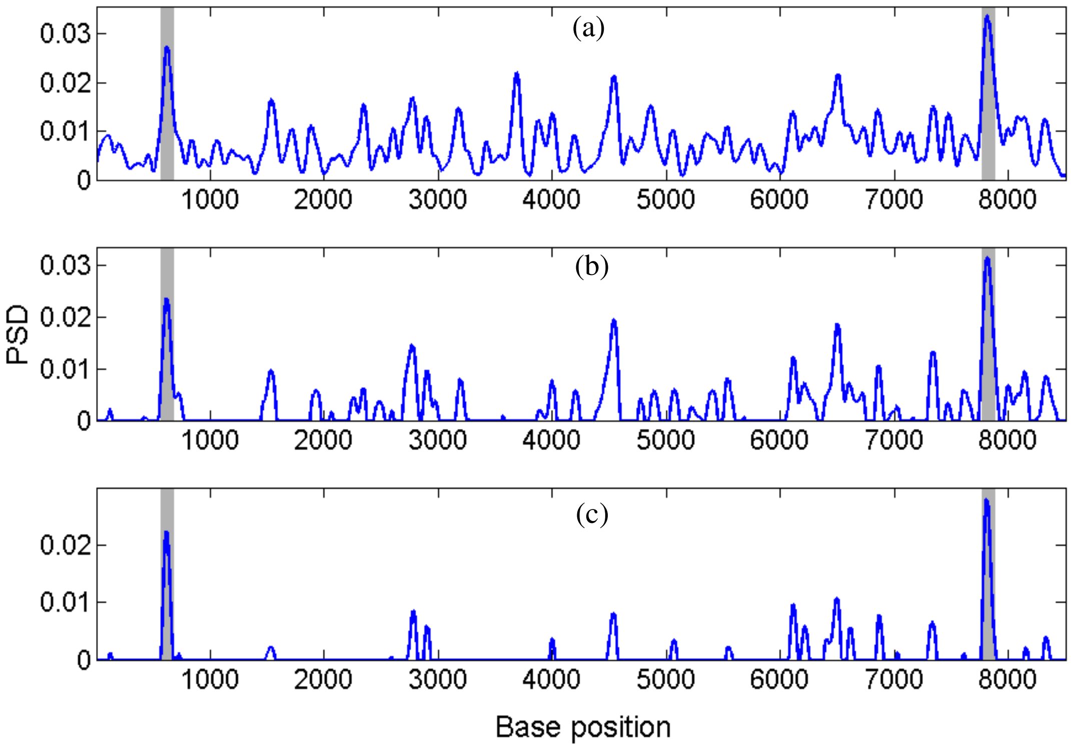

Figure S6.1 PSD plot for NW_003183000.1 (Callithrix jacchus) 537001 to 545500.

Figure S6.1 shows that subtraction of both 6bp and 9bp effects suppresses the noise. However, following plots show that true positive peaks can also be killed especially when 9bp effect is subtracted in addition to subtracting 6bp effects.

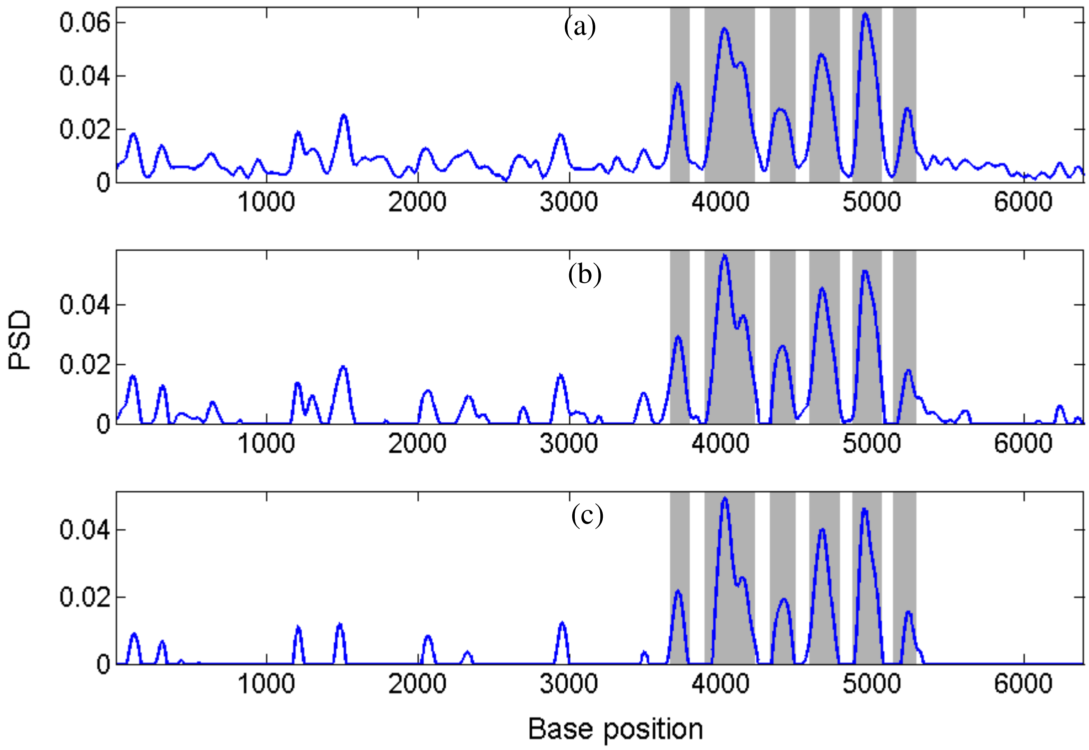

Figure S6.2 PSD plot for U02285.1 (Bos taurus alpha skeletal actin precursor gene).

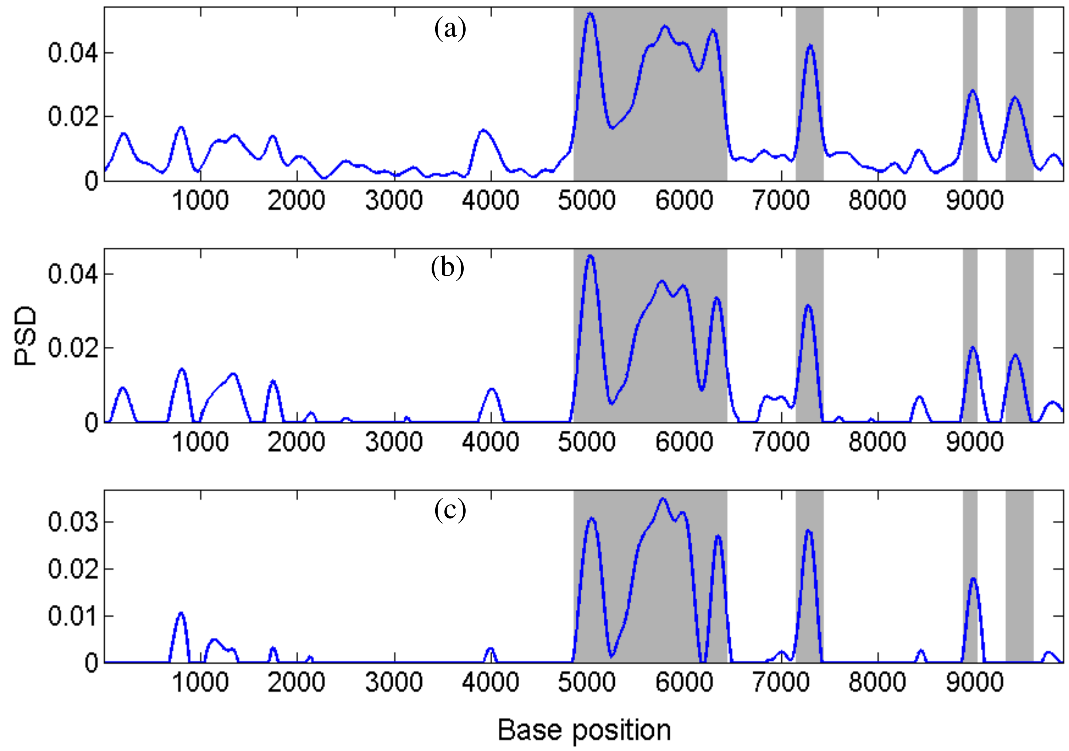

Figure S6.3 PSD plot for sequence X78212.1 (H.sapiens diamine oxidase gene).

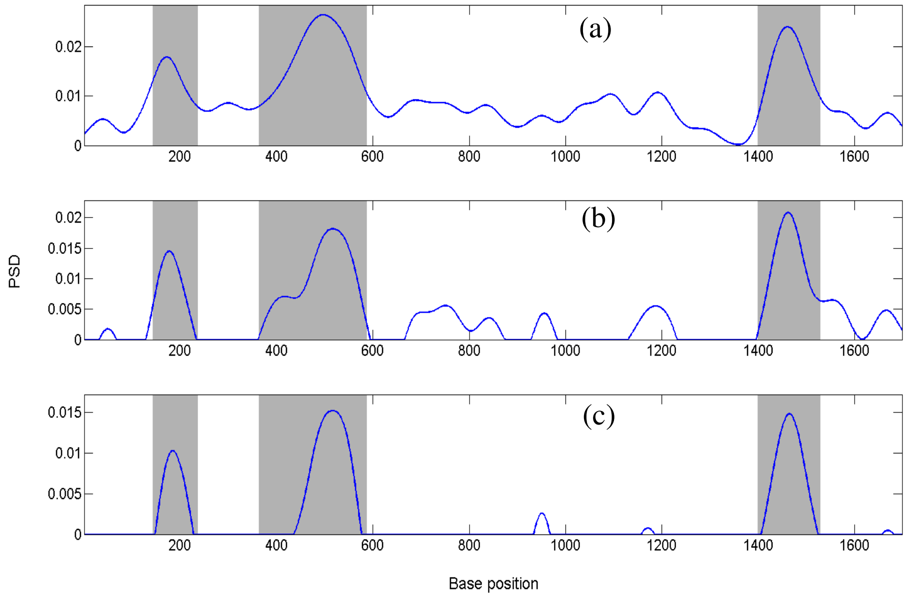

Figure S6.4 PSD plot for L25361.1 (Cebuella pygmaea epsilon-globin gene).

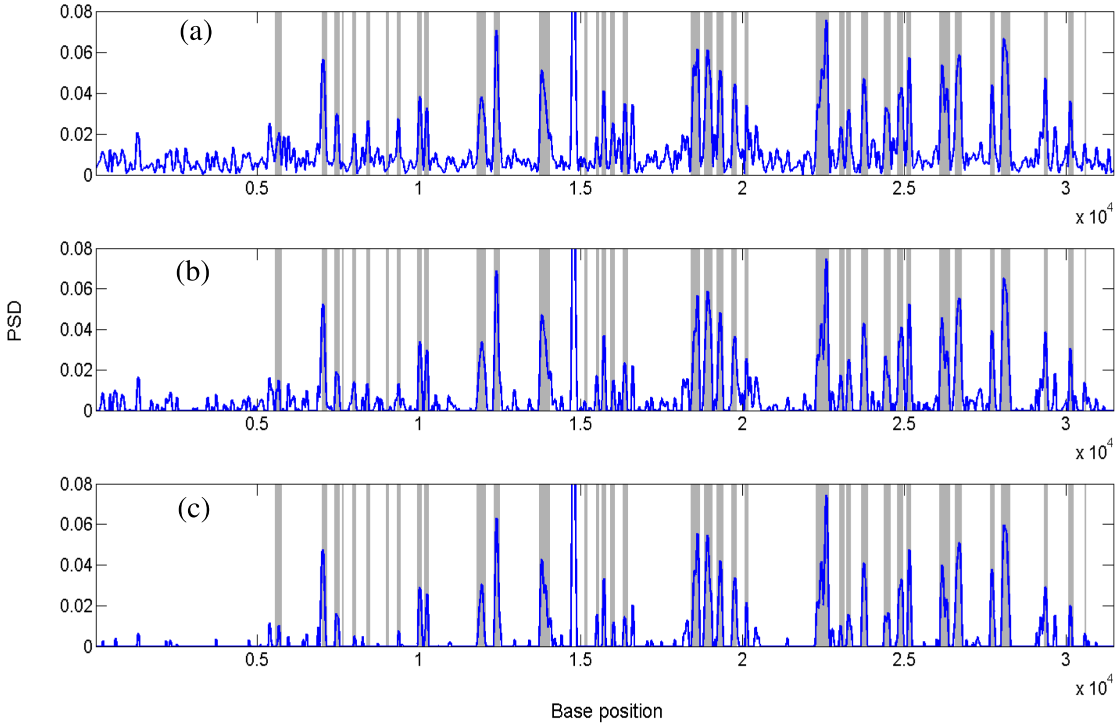

Figure S6.5 PSD plot for Z20656.1 (H.sapiens gene for Me491/CD63 antigen).

Table S1. Summary of TP signal for human exons under various base deletions

45 human exons are randomly picked for comparison of TP maximum amplitude with 1-base deletion at various base positions, and then followed by 2-base deletion. Type 1, 2 or 3 may overlap with each other (observing both).

Type	Description	Frequency
1	No likely evolutionary frameshift<br>(Diminished by 1 bp deletion, restored by subsequent 2 bp deletion)	35/45
2	Likely 2 bp evolutionary frameshift (TP Enhanced by 1 bp deletion)	6/45
3	Likely 1 bp evolutionary frameshift (TP Enhanced by 2 bp deletion)	5/45
4	Ambiguous pattern	5/45
